# Aiming for the Sweet Spot: Glyco-Immune Checkpoints and γδ T Cells in Targeted Immunotherapy

**DOI:** 10.3389/fimmu.2020.564499

**Published:** 2020-09-29

**Authors:** Margarita Bartish, Sonia V. del Rincón, Christopher E. Rudd, H. Uri Saragovi

**Affiliations:** ^1^Lady Davis Institute, Jewish General Hospital, Translational Center for Research in Cancer, McGill University, Montreal, QC, Canada; ^2^Oncology and Experimental Medicine, McGill University, Montreal, QC, Canada; ^3^Division of Immuno-Oncology, Research Center Maisonneuve-Rosemont Hospital, Montreal, QC, Canada; ^4^Département de Médecine, Université de Montréal, Montreal, QC, Canada; ^5^Pharmacology and Therapeutics, and Ophthalmology and Vision Sciences, McGill University, Montreal, QC, Canada

**Keywords:** gangliosides, sialic acid, tumor marker ganglioside, γδ T cells, immunotherapy, cancer

## Abstract

Though a healthy immune system is capable of recognizing and eliminating emergent cancerous cells, an established tumor is adept at escaping immune surveillance. Altered and tumor-specific expression of immunosuppressive cell surface carbohydrates, also termed the “tumor glycocode,” is a prominent mechanism by which tumors can escape anti-tumor immunity. Given their persistent and homogeneous expression, tumor-associated glycans are promising targets to be exploited as biomarkers and therapeutic targets. However, the exploitation of these glycans has been a challenge due to their low immunogenicity, immunosuppressive properties, and the inefficient presentation of glycolipids in a conventional major histocompatibility complex (MHC)-restricted manner. Despite this, a subset of T-cells expressing the gamma and delta chains of the T-cell receptor (γδ T cells) exist with a capacity for MHC-unrestricted antigen recognition and potent inherent anti-tumor properties. In this review, we discuss the role of tumor-associated glycans in anti-tumor immunity, with an emphasis on the potential of γδ T cells to target the tumor glycocode. Understanding the many facets of this interaction holds the potential to unlock new ways to use both tumor-associated glycans and γδ T cells in novel therapeutic interventions.

## Introduction

The cornerstone of a healthy immune system is the ability to distinguish “self” from “nonself,” to mount a response to “nonself” while minimizing the reactivity to “self” (1). A tumor originates from cells that remain mostly “self.” Thus, identifying meaningful differences between pathological and healthy cells has been difficult in the dynamic tumor microenvironment (TME). Nonetheless, the erratic pattern of gene expression, altered metabolism, deregulated signaling pathways, and often high mutational burden results in the presentation of neoantigens on the surface of tumor cells. These novel antigens can be recognized by both the innate and adaptive arms of the immune system, although this response can be counteracted by the TME via immunoediting, immunoevasion, and immunosuppression. For example, tumors can exploit inhibitory receptors on T-cells by inducing an unresponsive or exhausted state (2).

Research in the area of novel immunotherapies has been focused on deploying, re-educating or enhancing immune defenses to overcome the suppressive, detrimental TME. The past decade has witnessed a revolution in the application of immunotherapy for the treatment of cancer, resulting in the approval of immune checkpoint blockade (ICB). ICB uses monoclonal antibodies (mAbs) directed against the inhibitory receptors (IRs) present on the surface of T cells, or the natural ligands of IRs, often expressed by cancer cells. The blockade of IR-ligand interactions reduces the inhibitory regulation of T cells. ICB against cytotoxic T-lymphocyte–associated antigen 4 (CTLA*-*4) and programmed death 1 (PD-1) or the PD-1 ligand (PD-L1) have produced survival benefits for an ever-expanding list of malignancies (3). However, only a limited subset of patients benefit from ICB, and there are, at times, toxic side effects due to inflammation and autoimmunity (4). Thus, there is a need to understand the mechanisms used by cancer cells to suppress and shape immune responses and to involve novel immune cell subsets in the design of anti-tumor targeted therapies. Here, we discuss the role of glycans in the context of immunity.

The changes in gene expression that accompany malignant transformation have a significant impact on the glycome, glycoproteome and glycolipidome—the glycocode of cancer cells—leading to the overexpression and *de novo* expression of novel glycan epitopes (5). These have been studied extensively in the context of promoting tumor cell-intrinsic aspects of proliferation, signaling and metastasis. Relatively recently, the glycocode of tumor cells has been implicated in suppressing anti-tumor immunity, emerging as a novel immune checkpoint, and, thus, a target for immunotherapy. While now recognized as an axis of immune modulation with druggable and therapeutic potential (6), its potential has remained underdeveloped clinically. Moreover, the subset of immune cells that attack carbohydrate targets remains poorly understood. In this review, we discuss the way in which γδ T cells have the potential to become effectors against carbohydrate moieties on cancer cells.

## Glycosylation in the Tumor-Immune Cell Interplay

All cells are covered with a dense coat of glycans, chains of carbohydrates that are covalently attached to proteins or lipids (7). Glycan diversity is immense, stemming from the numerous monosaccharide building blocks that can be assembled into linear or branched chains of various lengths by multiple types of chemical bonds, and diversified further by coupling to proteins, nucleic acids or lipids (8). This diversity creates a unique glycan “landscape” of expression for each cell and constitutes a major aspect of the molecular interface between cells and their environment. Glycans are also important for the transport of nascent proteins to the surface of cells as well as, in a larger context, the maintenance of tissue structure and extracellular matrix organization, cell membrane integrity, cell-cell adhesion, and cellular signaling. To immune cells, surface glycans serve as an identifying feature of a cell, a calling card of sorts (9, 10).

Aberrant glycosylation is a hallmark feature of cancer cells (11–13). Key among the distinguishing features of a tumor's “glycan topography” is the anomalous expression of sialic acid–carrying glycans (sialoglycans) (14). Sialic acids are a family of negatively charged, nine-carbon sugar molecules linked to mucins, extracellular matrix, cell surface glycoproteins (N- and O-linked oligosaccharide chains), or glycolipids by α-2,3; α-2,6 and α-2,8 linkages (15).

Tumor cells are covered with a dense layer of sialoglycans, some of which are uniquely associated with malignancy (16). This coating protects tumor cells from being recognized and eradicated by the immune system, as it can both mask their “non-self” immunogenicity and interfere with immune cell function (17, 18). For instance, elevated sialylation of cancer cells disrupts the interaction of the NK-activating receptor natural killer group 2D (NKG2D) with ligands on the tumor cells, reducing NK-activating signals derived from tumor cells (19). This strategy by tumor cells is reminiscent of sialic acid coatings used by parasites and other pathogens to evade immunity (20). Despite these examples linking protein sialylation to pathology, we note that this post-translational modification is not always deleterious. Sialylation of some proteins is associated with neuroprotective signals (15).

### The Sialic Acid-Siglec Axis of Tumor Immunomodulation

As “self-associated molecular patterns” (SAMPs), sialic acids are recognized by sialic acid-binding Ig-type lectins (Siglecs). Twenty years of study document the importance of sialic acids in discriminating “self” and “non-self,” showing the existence of natural antibodies to a variety of sialidase-treated immune cells in human serum [reviewed in (21)]. In humans, the Siglec family comprises 14 members. These are subdivided into the conserved Siglecs:−1 (Sialoadhesin/CD169),−2 (CD22),−4 (Myelin-associated glycoprotein/MAG),−15, and the CD33-related Siglecs−3,−5 to−11,−14 and−16 (22). The Siglecs are composed of modular immunoglobulin-like (Ig-like) domains, usually with the V-like domain at the N-terminus mediating binding to sialic acids. This domain shows a high degree of sequence similarity to other Ig-like domains in the receptor family with the exception of the C-2 set Ig domains near the plasma membrane. The cytoplasmic domains have immunoreceptor tyrosine-based inhibition motifs (ITIMs) that bind to the protein tyrosine phosphatases src homology region 2 domain-containing phosphatases 1 and 2 (SHP-1 and SHP-2). SHP-1 has a clear negative signaling role, while SHP-2 has been shown to play both positive and negative roles in immune cells.

Functionally, Siglec binding to sialic acid facilitates tolerance to cell membrane antigens expressed by the same cell. In B cells, for example, Siglec-sialic acid binding suppresses B cell activation and stimulates B cell apoptosis (23–25). While a key physiological mechanism to prevent autoimmunity, inhibitory Siglec-sialic acid interaction illustrates how an immunological fail-safe can be hijacked by tumors to escape host immunity. The engagement of Siglec-7 and Siglec-9 on NK cells by tumor-associated sialic acids inhibits NK cell activation (26–28). Conversely, the loss of Siglec-7 expression on an NK cell line promotes sustained cytotoxic activity against leukemia cells *in vitro* (29). Furthermore, the binding of the cancer-associated sialylated glycoform of MUC1 to Siglec-9 on macrophages resulted in their differentiation to immunosuppressive so-called M2 macrophages with upregulated PD-L1 expression (30). Tumor-associated macrophages were also shown to express Siglec-15, and the interaction between Siglec-15 on M2 macrophages and tumor-associated sialyl-Tn (sTn) antigen elevated macrophage-produced TGF-β, a known pleiotropic mediator of pro-tumor responses (31). Recently, tumor-expressed CD24 (a highly sialylated glycoprotein) was shown to hinder the ability of tumor-associated macrophages to phagocytize tumors via binding to Siglec-10 (32). The expression of multiple members of the Siglec family was shown on myeloid-derived suppressor cells (MDSCs) of glioma patients, although the functional consequences of this expression on this already immune suppressive cell type are unclear (33).

The innate and adaptive arms of the immune system can influence each other, thus the effects of sialoglycan-Siglec interactions may indirectly affect the activation status and function of the cells of adaptive immunity. For example, the interaction between sialylated antigens and Siglec-E (murine ortholog of human Siglec-9) on dendritic cells can influence the T cell population, favoring differentiation of antigen-specific regulatory T cells and reducing the numbers of effector T cells (34). Although the expression of Siglecs on normal T cells is low, these can become elevated in tumor-infiltrating lymphocytes (TILs), resulting in suppressed anti-tumor T cell function (35, 36). In melanoma, Haas et al. identified Siglec-9 expression on tumor-infiltrating, but not peripheral, CD8^+^ cells (36). By disrupting the sialoglycan-Siglec pathway between tumor cells and T cells, Stanczak et al. demonstrated a delay of tumor growth and an increased infiltration of CD8^+^ T cells in a mouse model of colorectal cancer (35).

Given the role of tumor sialylation in the establishment of an immunosuppressive TME, both sides of the sialoglycan-Siglec axis have been targeted therapeutically. To reduce the sensitivity of immune cells to tumor sialoglycans, Siglec function can be blocked with monoclonal antibodies (mAbs). MAbs have been tested pre-clinically against Siglec-7 (26), Siglec-9 (35), and Siglec-15 (37). A challenge of this approach is to confine the response to the tumor setting, as deregulated immune activation might have detrimental consequences outside of TME. The expression of Siglec-9, for example, is restricted to TILs, thus its systemic inhibition by a mAb is unlikely to impact peripheral cytotoxic CD8^+^ T cell function. However, Siglec-9 is also abundantly expressed by neutrophils and a blocking antibody could result in their uncontrolled and potentially damaging activation. An alternative direction is to target the causative agents of immunosuppression, namely sialoglycans on the tumor cells, rather than on receptors of immune cells. Stalling *de novo* sialic acid synthesis, via the use of glycomimetic sialic acid analogs that cannot be attached to the glycan chain, has been shown to reduce the density of sialoglycans on the tumor surface and delay of tumor growth and metastasis (38, 39).

Finally, the sialoglycan coverage on tumor cells might be “shaved” using sialidases, sialic acid trimming enzymes. In the 1960s and 1970s, it was found that injecting a tumor-bearing animal with growth-arrested tumor digests treated with sialidases—a very early form of cancer vaccination—impeded the growth of the pre-existing tumor in mouse (40) and dog (17) cancer models (41). Despite promising results in early trials on advanced patients using sialidase-treated cancer cells to boost the immune response, this form of immunotherapy did not become a standard of care (42, 43). In recent years, in an approach the authors termed precision glycocalyx editing, a pre-clinical study coupled a recombinant sialidase to a therapeutic mAb against the human epidermal growth factor receptor 2 (HER2) (44). The antibody directed the effects of the sialidase to the HER2-expressing tumor cells, simultaneously reducing Siglec-mediated NK cell suppression and exposing the tumor cells to NK cell-mediated antibody-dependent cytotoxicity.

## Gangliosides as Part of the Tumor “Glycocode”

Among the key sialic acid-containing glycocompounds found on the surface of tumors are the gangliosides—a family of glycosphingolipids with one or several sialic acid molecules attached to the extracellular carbohydrate chain. Though named after the cell type from which they were first isolated— “Ganglienzellen,” neurons—gangliosides are ubiquitously expressed on the membranes of all eukaryotic cells, typically clustering in cholesterol-rich lipid microdomains or rafts (45). Indeed, evidence suggests that gangliosides co-localize with signaling molecules and adhesion molecules in glyco-signaling domains on the cell surface (46).

It is the unique glycan tree-structure that defines each different ganglioside. In ganglioside nomenclature, the prefix G stands for “ganglio” while the letters M (mono-), D (di-), T (tri-) and Q (quad-) denote the number of sialic acid molecules. Further classification is made on the basis of thin layer chromatography migration and is represented by Arabic numerals and lower case letters reflecting the order of migration of each corresponding type (47). GM1 is expressed on most eukaryotic cells and has a prominent role in the activation of intracellular signals in neuronal and lymphoid cells. In particular, GM1 represents the major ganglioside component of the brain (48), with several key neuronal functions becoming compromised as a consequence of decreasing GM1 levels during, for example, aging (49, 50). In contrast, GD2 and GD3 are almost exclusively expressed in tumor cells (51). As such, GD2 and GD3, are examples of a subset of gangliosides referred to as tumor marker gangliosides (TMGs), a family comprising about 20 different gangliosides present preferentially or almost exclusively and at high density on the cell surface of certain cancers (Table 1).

**Table 1:** Tumor marker ganglioside targets.

**Malignancy**	**GD2**	**GD3**	**Fucosyl-GM1**	**GM2**	**GM3**	**PolySia (52)**	**Sialyl Lewis X**
Neuroblastoma	(53–55)	(55)	(56)	(57, 58)	(59)	(60, 61)	
Melanoma	(55)	(55, 62)	(63)	(64)	(65)		
Glioma	(66)	(67–69)		(52, 70)	(71–73)	(74)	
Non-small cell lung cancer (NSCLC)			(75)	(76)	(77, 78)	(79, 80)	(81, 82)
Small cell lung cancer (SCLC)	(83)	(83)	(75, 83, 84)	(83)			
Breast carcinoma	(85)	(85–87)		(85)	(85)	(88)	(81, 82)
Renal cell cancer			(89)	(90)	(91)		(92)
Ovarian cancer	(93)	(94)	(93)	(93)	(95)		
Soft tissue sarcomas	(96)	(96)					
Osteosarcoma	(97)	(98, 99)					
Ewing's sarcoma	(100, 101)	(99)			(102)		
Desmoplastic Round Cell	(103)	(99)					
Rhabdomyosarc.	(99)	(99)					
Retinoblastoma	(104)				(105)		
Wilms tumor					(91, 106)	(107)	
Medullary thyroid cancer						(108)	
Prostate Cancer				(109)			
Gastric cancer				(109)		(109)	(81)
Endometrial				(109)		(52, 109)	
Pancreatic				(109)		(109)	(81)
Colon Cancer				(109)			(81, 82)
Esophageal							(81)
Head and neck							(81, 82)

*Select cancers where there is evidence for TMG expression in >50% of all patients in the indicated malignancy. This table is shown to exemplify the prevalence of TMGs. The cells with no entry reflect ≤ 50% prevalence, or that we omitted literature that we deem unreliable for this review because very few biopsies were phenotyped. The list ranges from ~95% (Neuroblastoma), to ~80% (Melanoma), to ~50% (Head and Neck) of patients. When expressed in a patient the TMGs are present homogeneously in tumor nodules and cells. Gold color denotes literature from many laboratories, or evidence confirmed by the authors of this review*.

Ganglioside biosynthesis begins in the endoplasmic reticulum, with the synthesis of the ceramide precursor, common to all glycosphingolipids, and continues in the Golgi apparatus where the ceramide is converted to glucosylceramide. Sugar residues—galactose, glucose and sialic acids—are added, one by one, catalyzed by specific glycosyltransferases. Some gangliosides can also result from the removal of a sugar or sugar branch by glycosidases. As several enzymes or pathways can generate a ganglioside, their biosynthesis is hard to target. Nonetheless, this strategy has been explored in pre-clinical studies. The inhibition of glucosylceramide synthase, the enzyme which catalyzes the first step in glycosphingolipid synthesis, by N-butyldeoxynojirimycin (NB-DNJ) has been shown to temporarily delay tumor onset in a mouse melanoma model (110). However, prolonged treatment with NB-DNJ is toxic, and in the absence of the inhibitor, ganglioside levels rapidly recovered. Targeting GD3 synthase, the enzyme responsible for the biosynthesis of GD2 and GD3, reduced tumor stem cell functionality, abrogated *in vivo* tumor formation (111), and interfered with the epithelial-to-mesenchymal transition and metastasis in murine models (112).

Upon export to the plasma membrane, the sphingolipid (ceramide) part of the molecule—two lipid tails consisting of the long-chain amino alcohol sphingosine coupled to a fatty acid—anchors the ganglioside to the cell surface, while the glycan moiety is exposed to the external environment. Apart from the plasma membrane, their main cellular location, gangliosides are also detected in other cellular organelles, including the nuclear envelope (113) and mitochondria (114). Importantly, they can also be actively “shed” and taken up by other cells (115). The excretion of gangliosides from a cell into the extracellular environment is poorly studied, and may be the result of release in the form of exosomes, microparticles, or as micelles given the physical properties of the gangliosides (hydrophobic tail and hydrophilic head). Extracellular gangliosides improve tumorigenicity of poorly tumorigenic cells in mouse models (116) and have been implicated as a mechanism by which tumors suppress immune cell function (117, 118).

### Biology and Function of Tumor Marker Gangliosides

As the term “tumor marker” suggests, TMG expression is tightly associated with malignant cells. Table 1 summarizes the cancer types with a high TMG prevalence across patients. However, in addition to their status as biomarkers of malignancy, GD2 and GD3 play active roles during cancer development, with proven links to tumor growth, metastasis, and immune evasion.

Because of their accumulation on the outer leaflet of the plasma membrane (with the sugars being extracellular), gangliosides participate in cellular communication. The carbohydrate “head” of gangliosides can interact with proteins, lipids, and glycans present in the extracellular matrix and on other cells. It can also interact laterally within the membrane to regulate lipid rafts or microdomain formation (119). As components of rafts, gangliosides affect signaling processes during cancer progression. For example, GD2 and GD3 promote ligand-independent activation of wild type receptor tyrosine kinases (RTKs), including EGF-R, TrkA, TrkB, PDGF-R, IGF1-R, MET, as well as cytoplasmic *src*-related kinases (e.g., Src, Lck) (98, 120, 121). The expression of GD2 and GD3 can therefore be viewed as pro-oncogenic and may be etiological in tumors where oncogenic mutations are not clearly identified.

The pro-tumor roles of tumor gangliosides have been implicated at all stages of tumor development. GD2 expression was linked to breast cancer stem cell phenotypes, while suppression of its biosynthesis in breast cancer cell lines decreased mammosphere formation and tumor initiation (111). *In vitro* experiments have connected ganglioside production to an increase in cancer cell migration and invasion (98). Conversely, a mAb against GD2 was shown to induce apoptosis in small cell lung cancer cells (83, 122) and an anti-GD3 antibody inhibited the growth of human melanoma cells *in vitro* (123). Cancer cells devoid of GM2 and GM3 synthases formed avascular tumors, suggesting the involvement of TMGs in angiogenesis during tumor growth (124). Additionally, the exogenous addition of GD3 to glioma cells stimulated VEGF production, suggesting a role for tumor-shed gangliosides in *de novo* blood vessel formation (125). In patient-derived melanoma cells, ganglioside expression is tightly linked to melanoma aggressiveness and patient survival, with patients expressing GD3 having the shortest survival (126).

Building on the discussion of glycan-containing sialic acids as a segment of the tumor glycocode used by the tumor to evade anti-tumor immunity, GD2 and GD3 can be regarded as immunosuppressive, even in cases where other immune escape proteins such as PD-1 or PD-L1 are absent. Indeed, the observations that TMGs can inhibit antibody production and lymphocyte proliferation were first made decades ago (127, 128). This is increasingly relevant in modern immunotherapy as it is possible that the high failure rate of conventional ICB therapy in melanoma (targeting PD-1 or PD-L1) is associated with high GD2/GD3 expression, a hypothesis that we are evaluating experimentally. As sialic acid-containing compounds, GD2 and GD3 can interact with Siglecs (129–131). Siglec-7, in particular, displays a strong affinity for the α2,8-linked disialic acids found on GD2 and GD3 (132). Additionally, TMGs influence the recruitment and function of immune cells in Siglec-independent ways. TMGs interfere with IL-2/IL-2R binding, key to T cell proliferation (133). They have also been shown to induce apoptosis in T cells (90) and dendritic cells (134), and impair antigen presentation in human monocytes (135). In a tumor model engineered to lack GM3, GM2, GM1, and GD1a, the observed impairment of tumor growth was attributed to a reduction, and decreased activity, of MDSCs (136). Intriguingly, the presence of MDSCs could be restored by exogenous supplementation of gangliosides which suggests a direct connection between tumor-produced gangliosides and the recruitment of immunosuppressive MDSCs to the TME.

### TMGs as Therapeutic Targets

In 2009, GD2 was “ranked” by the NCI as 12th in priority of all clinical cancer antigens, with additional three gangliosides (GD3, fucosyl-GM1, and N-acetyl GM3) included in the list of 75 prioritized antigens (137). The high expression of GD2 and GD3 in cancer makes these promising targets for therapeutic intervention. Moreover, when GD2 or GD3 are present in a cancer, tumor cells express them stably and homogeneously, and tumor microheterogeneity with regards to TMG expression has not been reported. GD2 or GD3 persist throughout tumor progression, and expression does not appear to downregulate after chemotherapy, in at least the reported studies of neuroblastoma (138), osteosarcoma (139), and in the *ex vivo* examination of several cell lines (140).

The etiological role of GD2/GD3 in oncogenesis and immune suppression are additional features that would make these glycolipids ideal therapeutic targets for clinical translation. However, exploiting GD2 or GD3 has been challenging. Monoclonal antibodies against GD2 [Dinutuximab/Ch14.18 mAb (141–143) and 3F8 mAb (144)] and GD3 [BEC2 (145), R24 (146)] achieved partial success in cancer therapy as passive immunity (i.e., the administration of purified antibodies against a target). However, they cause serious adverse effects, such as high-grade visceral pain, that is not blocked by morphine (147). While Ch14.18 mAb, in combination with GM-CSF and IL-2, stimulates antibody-dependent cell-mediated cytotoxicity and improves overall survival in neuroblastoma (141), in clinical trials it had low efficacy or exhibited a low therapeutic index [reviewed in (148)]. More recently, engineered chimeric antigen receptors (CAR) expressing an anti-GD2 mAb sequence in T or NK cells, were used in combination with ICB inhibitors and cytokines (149), but the cells did not persist in circulation and the treatment showed no efficacy (150). The failure is not surprising given that the CAR was engineered from mAbs that also exhibit a low therapeutic index in passive immunity. In addition to the clinical CAR T cell studies performed in neuroblastoma and melanoma, pre-clinically, CAR anti-GD2 T cells have recently been tested against breast cancer (151) and diffuse midline glioma (152).

Historically, the development of anti-GD2 or anti-GD3 vaccines has been tried without success. Glycolipids are poorly immunogenic and are not thought to be processed by antigen presenting cells or presented by MHC antigens. While lipids can be recognized by specialized CD1 (MHC class I-like molecules)–restricted T cells, each ganglioside does not have a unique type of lipid. In fact, the lipid tails can be heterogeneous within a single ganglioside (ranging in carbon chain length, oxidation and saturation state). Hence, only subtle differences exist between normal GM1 and tumor GD2 and GD3 carbohydrate heads, and their lipid tails can be shared or can exhibit heterogeneity across all gangliosides whether they are normal or TMGs. Thus, when using whole TMGs as immunogens, there are concerns with regards to tolerance, cross-reactivity or transient and ineffective immune reactions.

Notwithstanding the aforementioned concerns, initial attempts at developing GD2/GD3 vaccines used native GD2 or GD3 glycolipids chemically conjugated to carriers (153, 154). These were somewhat immunogenic and induced humoral responses that delayed tumor growth in mice via a complement-dependent cytotoxicity (CDC) mechanism. However, anti-ganglioside antibody titers were not long-lasting even after multiple immunizations, and there was no correlation between humoral titer and tumor therapeutic efficacy (147).

## The Potential of γδ T Cells in Targeting the “Glycocode”

γδ T cells, expressing the gamma and delta chains of the T-cell receptor (TCR) coupled to the CD3 invariant signaling chains, are a subclass of T lymphocytes whose defining characteristic is their ability to display traits of both the innate and adaptive immune systems (155). They express a TCR whose engagement with its target mediates T cell activation. Human γδ T cell subsets—the subsets in other species will differ—are classified according to the Vδ chain in the TCR. Vδ(1-8) together with one of 6 different Vγ chains (2–5, 8, and 9) forms the mature TCR via V(D)J recombination. In this manner, they generate TCR diversity similarly to conventional αβ T cells.

On the other hand, the TCRs of the γδ type recognize qualitatively distinct antigens, with kinetics, antigen recognition mechanisms and tissue localization fundamentally distinct from αβ T cells. While some γδ T cells can found in circulation, with the Vγ9Vδ2 being the major subset corresponding to about 5% of total CD3^+^ cells in the periphery (156), the two other main γδ T cell subsets, Vδ1 and Vδ3, are predominantly tissue-resident. Like innate immune cells, γδ T cells recognize targets with broad patterns of pathogen-encoded or dysregulated-self signatures, as opposed to the specificity displayed by the αβ T cells.

### Antigen Recognition by γδ T Cells

Unlike αβ T cells, γδ T cells do not rely on peptide presentation by the MHC complex of antigen-presenting cell to become activated. The precise mechanisms behind γδ T cell antigen recognition remains a field of intense research, complicated by the vast array of structurally diverse classes of self and non-self-ligands recognized by the γδ TCR (157). This includes soluble and membrane-bound proteins and peptides of a wide range of sizes, as well as non-protein targets such as phospholipids, non-peptidic antigens and carbohydrates.

Although often referred to as MHC-unrestricted, some of the most well-characterized targets of γδ-TCR include the non-classical class I MHC molecules. The MHC-I related molecules T10 and T22, found specifically in mice, were the first ligands whose binding to the γδ-TCR was confirmed biochemically (158, 159). Recognition of lipids presented by the CD1 family of MHC class I-like proteins was established a few years later (160–162) and is a key aspect of TME-related γδ T cell biology. Vδ1 and Vδ3 T cells can recognize the sphingolipid α-galactosylceramide (α-GalCer) presented by CD1d and, as a consequence, upregulate cytokine production characteristic of Th0 (i.e. IFNγ and IL-4), Th1 (i.e., IFNγ) and Th2 (i.e., IL-4) cells (163, 164). Recently, a third type of non-classical class I MHC molecule, the MHC-related protein 1 (MR1), was shown to be a target of γδ-TCR (165). This protein is involved in the presentation of microbial metabolites related to vitamin B2 biosynthesis and is known to stimulate a special subset of αβ T cells known as mucosal-associated invariant T cells (MAIT cells). Intriguingly, the resolved crystal structure of a γδTCR–MR1–antigen complex revealed a key difference to the previously proposed modes of TCR-ligand recognition. The γδ TCR was found to bind underneath the MR1 antigen-binding cleft, suggesting a new “antigen-agnostic” mode of TCR-target interaction, the biological implications of which are not yet understood.

In addition to targets presented as a part of non-classical MHC molecules, several types of non-peptidic antigens have been described to activate T cells with γδ TCRs. Tumor cell recognition, in particular, is enhanced by the ability of γδ T cells to recognize such antigens, which often are by-products of dysregulated tumor processes (and which do not bind MHC molecules). Phosphoantigens, or phosphorylated non-peptide antigens, are the classical example of this principle. The phosphoantigen isopentenyl pyrophosphate (IPP) accumulates in tumor cells due to the deregulated mevalonate pathway (166), and specifically and potently activates Vγ9Vδ2 T cells (167). Although phosphoantigens were originally thought to activate the γδ TCR directly, new evidence shows that this recognition requires the participation of Ig superfamily family members known as the butyrophilins. In addition to the previously implicated butyrophilin 3A1 (168–170), two recent studies identified butyrophilin 2A1 as key for the recognition of phosphoantigens by γδ T cells (171, 172). Rigau et al. propose a model in which the phosphoantigen production by a target cell modifies a complex composed of butyrophilin 3A1 and butyrophilin 2A1 causing it to co-bind and activate the Vγ9Vδ2 TCR.

In addition to TCR receptor-mediated activation, it should be emphasized that γδ T cells also express other activating receptors, such as the NK cell receptors (173, 174). Building on an early study showing susceptibility to carcinogenesis in the absence of γδ T cells (175), Strid et al. showed that activation of NKG2D receptor by ligand Rae-1 (known as MICA in humans) on tissue-resident Vγ5Vδ1 γδ T cells inhibits skin cancer in a mouse model (176). In addition to skin, the role of NKG2D for γδ T cell activation was further shown in peripheral Vγ9Vδ2 cells (177). In NK cells, the NKG2D receptor has previously been mentioned in this text as being disrupted by elevated levels of sialylation on tumor cells. Given its now known functional roles in γδ T cells, it is thus possible that the immunosuppressive effects of elevated cancer cell sialylation can extend to impaired γδ T cell function.

### γδ T Cell Targeting of Carbohydrates in Tumor Gangliosides

The broad repertoire of targets recognizable by the γδ TCR as well as other receptors on the γδ T cells is suggestive of their potential use against specific carbohydrate targets on tumor cells, such as TMGs. However, this is an emergent field and concrete examples of such carbohydrate reactivity remain scarce.

The role of CD1d in γδ T cell activation provides an indirect example in which the spheres of γδ T cells and TMGs intersect. In one study, ovarian tumor-shed GD3 inhibited NKT-cell activation, with GD3 binding with high affinity to both human and murine CD1d. *In vivo* administration of GD3 suppressed α-GalCer-induced NKT cell activation in a dose-dependent manner, leading to the establishment of an immunosuppressive TME (94). While experimentally proven only in the context of NKT cells, it is possible that anti-GD3 blockade of the GD3-CD1d interaction would free up its recognition by, and subsequent activation of, γδ T cells, providing an additional therapeutic benefit. It should be noted that GD3-CD1d interaction can conversely have an immune activating effect. In melanoma, GD3 has been shown to activate NKT cells in a CD1d-dependent manner (178, 179).

Key to the potential of γδ T cells in targeting tumor-associated glycans is the fact that they do not require MHC presentation of antigens. This is relevant to tumor glycobiology because pure carbohydrates are typically not presented by MHC (180). While αβ T cells can recognize MHC-processed glycopeptides (peptides attached to a glycan), an early study determined the MHC-unrestricted carbohydrate specificity of γδ T cells (181). In addition, some MHC-restricted T-cell epitopes can be unaffected by glycosylation. An H-2Kb-restricted peptide retains an ability to be presented in its glycosylated form (hence, presentation is unaffected by peptide glycosylation) as the tethered carbohydrate fits in the central region of the TCR binding site. Hence, manipulation of γδ or αβ TCRs may yield previously unexploited strategies to target non-protein antigens in an MHC-unrestricted manner.

We recently reported the generation of synthetic GD2 and GD3 carbohydrate head-groups displayed on a multivalent polyamidoamine scaffold (PAMAM-GD2 and PAMAM-GD3). The PAMAM-GDs are lipid-free, water-soluble, inexpensive to produce, well-characterized chemically and structurally (including a crucial β-configuration at the first sugar), and identical to native carbohydrate head-groups on the surface of tumors (182, 183). These products (hereafter, called vaccines) are potent immunogens—when inoculated in mice, they stimulate B- and T cell immunity. The vaccines, as monotherapy, are therapeutic against four aggressive and metastatic syngeneic cancer models, significantly reducing primary tumors, metastatic burden, and importantly extending overall survival. Unexpectedly, this study revealed the expansion of γδ T cells mediated by a pure carbohydrate dendrimer. This occurs rapidly after vaccination in mice (independent of tumor presence) and in tumor-bearing mice (or upon tumor challenge) was followed by the expansion of the CD8^+^ T cells *in vivo*. Adoptive transfer of a relatively low number of the T cells isolated after vaccination is also therapeutic in tumor models (182).

**Figure 1:**
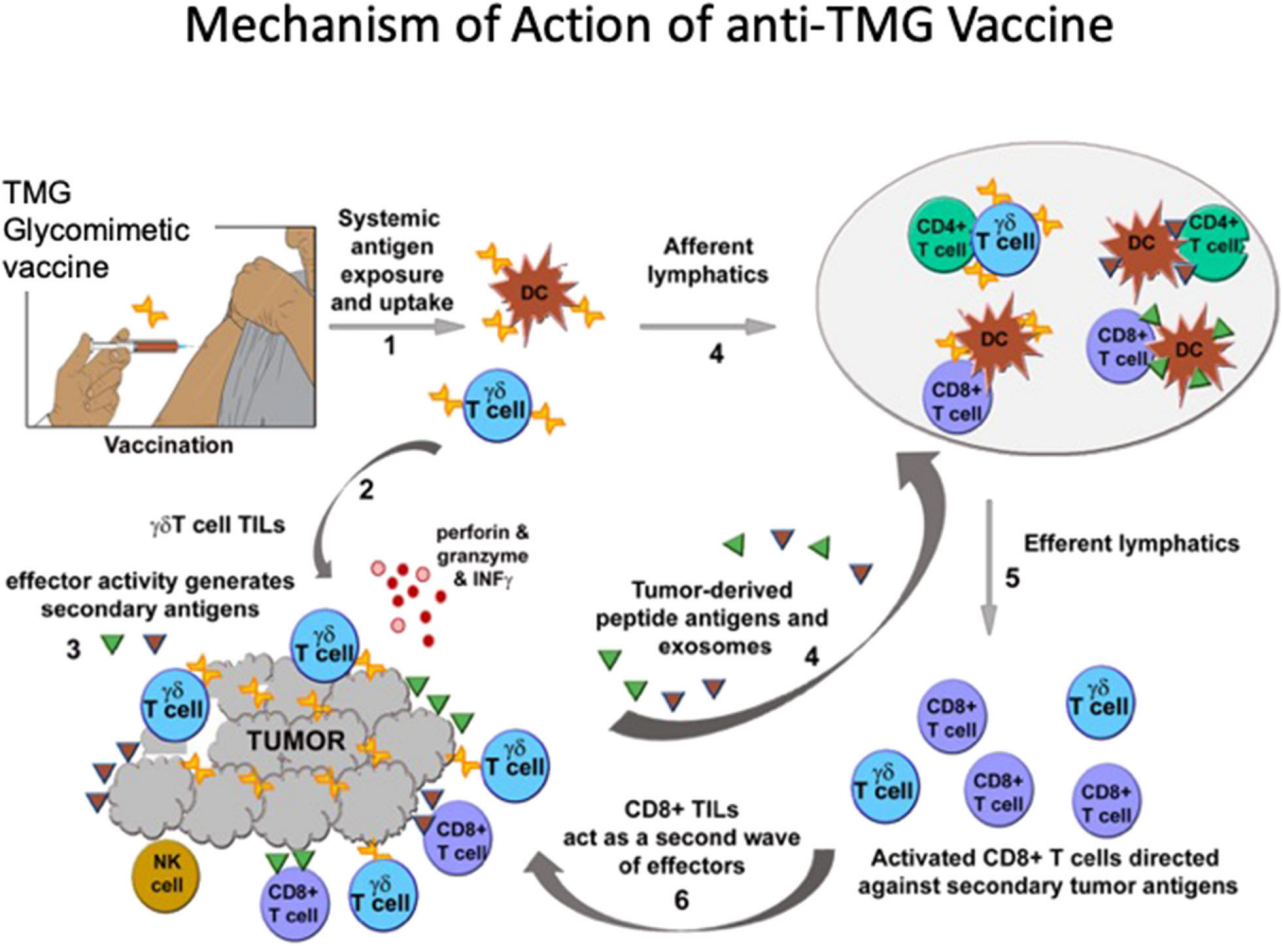
TMG glycomimetic vaccine mechanisms of immune activation. After systemic delivery of the vaccine antigens, (1) there is a rapid expansion of γδ T cells. It is unknown whether γδ TCRs expand by binding directly to the vaccine glycomimetic product, or whether the antigen is presented by DCs. Expansion of γδ T cells is independent of whether mice bear tumors expressing TMGs, so while the vaccine may also block the immune-suppressive action of TMGs upon T cells this mechanism is unlikely to account for the initial expansion. (2) In mice bearing TMG-expressing tumors, vaccination affords a significant increase in γδ T cells TILs. (3) The effector activity generates secondary antigens or neoantigens. (4) Putative neoantigens (not yet identified) circulate and are presented to CD8+ αβ T cells which (5, 6) expand as a second wave mainly comprising CD8+ T cells that also become TILs. The generation of *in vivo* T-cell memory in glycomimetic vaccines was not evaluated. However, anti-TMG humoral immunity (evaluated as a surrogate marker) matures and class-switches from IgM to IgG.

The data support the notion that vaccination can expand and activate γδ T cells directly (and perhaps through APCs), which then bypass the immunosuppressive TME and become TILs (Figure 1). Expansion of γδ T cells is detected in tumor-bearing as well as in non-tumor-bearing mice. Hence while the vaccine may also block the immune-suppressive action of TMGs upon T cells this mechanism is unlikely to account for the initial expansion, but may be relevant to anti-tumor efficacy. The initial expansion of γδ T cells is followed by a second wave of expansion and recruitment of CD8^+^ effector TILs. The ability of γδ T cells to activate other T cell subsets has been shown previously (184, 185). It is possible that the second wave of CD8^+^ effector TILs recognize neoantigens presented, or shed, by injured or stressed tumor cells. We note that while *in vivo* T-cell memory generated by glycomimetic vaccines was not evaluated, the anti-TMG humoral immunity matures and class-switches from IgM to IgG, and is a surrogate marker of memory.

The mechanism of action of the PAMAM-GD2 and GD3 vaccines and the role of γδ T cells in mediating immunity against TMGs is paradigm-shifting, because virtually all previous experimental and clinical data using vaccines directed against TMGs have focused on humoral immunity rather than on cellular immunity. Such bias was perhaps motivated by the early promising results of using anti-TMG mAbs as therapeutic agents.

The γδ T cells are susceptible to PD-1–mediated inhibition (186, 187), and the tumor models where the vaccines were evaluated express high levels of PD-L1. The high therapeutic efficacy suggests that the vaccines partially overcome the inhibitory effects of PD-L1 upon γδ T cells. Ongoing studies are evaluating whether combination therapy with ICB might augment the anti-cancer effects of vaccines.

Adoptive transfer of T cells isolated from vaccinated mice resulted in the appearance of γδ T cells as TILs, and in a high therapeutic index. However, the study did not evaluate the sequence of γδ TCRs that were expanded, and did not address whether the glycomimetic vaccine products bind directly to the γδ TCRs or are presented via CD1, for example. Moreover, the antigens and the mechanism causing a second wave of CD8^+^ T cell expansion, whether it is γδ T cell dependent, and whether it is relevant therapeutically, are key for the proper development of a future cancer vaccine. The TMG glycomimetic cancer vaccine is an exciting approach that requires further evaluation of immune-mechanisms and connections between TMG and γδ T cells. Also, it is noteworthy that the concept of harnessing γδ T cells and targeting sugars for cancer therapy has been under examination for non-cancer pathologies ranging from anti-viral, anti-bacteria, and anti-parasitic therapy (188–191) to autoimmune diseases (192).

## Therapeutic Targeting of γδ T Cells

The scientific literature regarding the clinical efficacy of γδ T cell therapy is overall positive, supporting the further exploration of their use in a clinical setting (156, 193). Studies performed thus far included patients with hematological malignancies (follicular lymphoma, multiple myeloma and acute myeloid leukemia), and non-hematological tumors, such as renal cell, breast and prostate cancer (194). Vδ1+ cells have shown promising results pre-clinically (195), and the infiltration of these cells correlated with necrotizing tumors and patient survival in melanoma (196). However, the bulk of clinical studies have used Vγ9Vδ2 T-cells due to their relatively high availability in the peripheral blood and their potential to be cultured, expanded and activated *ex vivo*. To activate the Vγ9Vδ2 T-cells, the butyrophilin-mediated reactivity of the Vγ9Vδ2 TCR to phosphoantigens can be exploited, using chemical compounds to elevate or mimic the expression of phosphoantigens either on tumor cells or on antigen-presenting cells in the TME. Such compounds include aminobisphosphonates (for example pamidronate and zoledronate) or synthetic phosphoantigen analogs (197, 198). The approach offers a useful tool for expansion, but is not necessarily a useful therapeutic approach, because the expanded Vγ9Vδ2 T cells are nearly monoclonal and are not specific for a desired antigen. Indeed, the clinical response in the trials conducted thus far has been minimal [reviewed in (199) and (200)]. Therefore, these experiments are an interrogation of γδ T cell biology, with some examination of safety parameters. Furthermore, the clinical data strongly point to the need for a combinatorial approach with other immune-based therapies for maximum efficacy (193, 201). Even with these limitations, the early results reported are encouraging (193).

### CAR γδT cell Therapy

T cells engineered to express chimeric antigen receptors (CARs) comprise a branch of immunotherapy that combines the antigen specificity of monoclonal antibodies with the signaling motifs of receptors to promote the proliferation of cytotoxic effector T cells. CAR therapy has been successfully applied in several types of hematologic malignancies (202–204), and while translation to solid tumors has been somewhat limited there is some success reported with CARs in breast cancer (151) and diffuse midline glioma (152).

It is possible that engineered CAR T cells, just as naturally occurring T cells, are restricted by the immunosuppressive TME. This could impair T cell recruitment, function, and survival. In this context, γδ CAR T cells are an intriguing alternative target. Transduction of γδ T cells with CARs might direct their cytotoxic activity specifically against a tumor antigen, while retaining their other advantageous features such as the ability to cross-present antigen to αβ T cells. Moreover, a key advantage of the non-MHC restricted nature of γδ T cells is that CAR γδ T-cell preparations can be generated and expanded from pooled healthy donors.

To maximize the efficacy of the CAR therapy, an ideal antigen for CAR generation would need to be tumor-specific, highly and stably expressed by all malignant cells, and etiological to tumor development. Highlighting the therapeutic potential of TMGs, one of the first studies to engineer γδ CAR T cells used the GD2-antigen (205). The authors reported that GD2-CARs of both Vδ1 and Vδ2 subsets were expanded in sufficient numbers for clinical studies. The expression of the GD2-CAR by γδ T cells enhanced their innate cytotoxicity by directing its effects against GD2-expressing tumor cells. Further amplifying the anti-tumor immune response, expanded CAR-transduced Vδ2 cells retained the ability to internalize and cross-present tumor antigens to αβ T lymphocytes.

γδ T cells were originally thought to lack memory, and this was a concern in the design of CAR γδ T cell therapies. A lack of memory potentially translates into short-lived anti-tumor responses, but this may be overcome by using the CAR γδ T cell therapy in multiple treatment cycles. Encouragingly, recent data from mouse and human studies suggest that γδ T cells indeed have characteristics reminiscent of memory αβ T lymphocytes, promoting antigen-specific adaptive immunity (206–209). For example, in mice infected with B. pertussis, lung resident memory γδ T cells were shown to expand upon secondary infection with increased production of the cytokine, IL-17 (207). In humans, Vδ1+ and Vδ2+ γδ T cells are evidenced to promote microbial-specific adaptive immunity (208, 209).

## Concluding Remarks

The concept of the “glycocode” (6, 210) poses that protein glycosylation—with sialic acids appearing to be key—regulates biological events that are crucial to immunity and cancer progression, comparable to, but beyond, the PD-1–type of checkpoint inhibition. This concept extends to the glycosylation of membrane and matrix proteins, mucins, and gangliosides, where TMGs represent a glyco-immune-checkpoint. Vaccines, antibodies, small molecules, soluble glycoproteins, enzymatic cleavage of sialic acids, or soluble competitors targeting the glyco-immune-checkpoint would be a promising approach to therapy (211).

It will be important to consider γδ T cell biology in the context of strategies targeting the glycocode and sialic acid-containing protein and ganglioside targets. Table 2 shows a comparison of the features of different cancer immunotherapies, including many that have a γδ T cell mechanism of action. Table 2 lists our view of their relative advantages and disadvantages. We present an overview of their current stage of development, which we view as a benchmark of the time that each approach has been in development (factoring time and investment of resources) and the degree of expectation of success. However, based on history, most approaches are expected to perhaps find a narrow niche or indication where they may be of utility. Unfortunately, most will either fail clinically, or will face difficult regulatory hurdles, or become untenable in the marketplace.

**Table 2:** Immunotherapeutic approaches involving γδ T cells or γδ TCRs.

	**Cancer immunotherapy approaches**		
**Features and mechanisms**	**Ganglioside Vaccines**	**mAbs, CAR-T, TILs, protein vaccines, antigen-pulsed dendritic cells, neoantigens**	**Therapeutic γδ T cells**
HLA-independent	Yes	No	Yes
Can present antigens	Yes	No/Poor	Yes/No
Can expand endogenous cytotoxic cells	Yes	No/Poor	Yes
Polyclonal responses	Yes	Yes in some, No in others	No/unknown
Genetic engineering required	No	Yes in some, No in others	Yes in some No in others
Target translates from animal models to humans	Yes	Often variable and model-or target-dependent	In progress
Validated targets	Yes	Yes in some, No in others	Yes/unknown
Target has known etiology in cancer	Yes	Yes in some, No in others	Yes/unknown
Invariant expression of tumor target	Yes	No	Unknown
Target expression may be quantified (personalized medicine)	Yes	Yes in some, No in others	No/unknown
Adjuvant or multiple dosing required	No	Yes	Unknown
Platform addressing multiple molecular targets	Yes	Yes in some, no in others	No
May be applied to multiple indications	Yes	Mostly no	No
Time to manufacture	Short	Long	Long
Manufacturing costs	Lower/low	High	High
Lymphodepletion or chemotherapy or cytokine treatment required	No	Yes	Yes/unknown
FDA regulatory hurdles	Lower	Higher	Higher
Examples of late preclinical or clinical development	Academic programs	Gritstone, Targovax, Gradalis, Agenus, Jounce, BioNTech, Neon, Precision Biologics, Vaccibody, Juno, Aurora, Triumvira, Adicet, Kite, etc	GammaDelta, Incysus, Gadeta, Lymphact, Immatics, etc

*The relative advantages and disadvantages of each are listed. The experience is evolving rapidly, and this list is only presented as an example, not meant to be comprehensive*.

Our work in developing glycomimetic vaccines surprisingly resulted in early activation of γδ T cells *in vivo*, and in high therapeutic efficacy in cancer. In addition to cancer, conceptually, this advance is applicable to therapies for other pathologies (e.g., antivirals) that could benefit from the activation of the innate and the adaptive immune systems by targeting sialic acids and other glycans.

## Author Contributions

All authors listed have made a substantial, direct and intellectual contribution to the work, and approved it for publication.

## Conflict of Interest

HS is inventor in patents for anti-TMG vaccines. The remaining authors declare that the research was conducted in the absence of any commercial or financial relationships that could be construed as a potential conflict of interest.
